# Non-Contact Oxygen Saturation Measurement Using YCgCr Color Space with an RGB Camera

**DOI:** 10.3390/s21186120

**Published:** 2021-09-12

**Authors:** Na Hye Kim, Su-Gyeong Yu, So-Eui Kim, Eui Chul Lee

**Affiliations:** 1 Department of AI & Informatics, Graduate School, Sangmyung University, Seoul 03016, Korea; 202032010@sangmyung.kr (N.H.K.); 202032015@sangmyung.kr (S.-G.Y.); 202032011@sangmyung.kr (S.-E.K.); 2 Department of Human-Centered Artificial Intelligence, Sangmyung University, Seoul 03016, Korea

**Keywords:** oxygen saturation, YCgCr color space, hemoglobin, Beer-Lambert law, photoplethysmographic signal

## Abstract

Oxygen saturation (SPO_2_) is an important indicator of health, and is usually measured by placing a pulse oximeter in contact with a finger or earlobe. However, this method has a problem in that the skin and the sensor must be in contact, and an additional light source is required. To solve these problems, we propose a non-contact oxygen saturation measurement technique that uses a single RGB camera in an ambient light environment. Utilizing the fact that oxygenated and deoxygenated hemoglobin have opposite absorption coefficients at green and red wavelengths, the color space of photoplethysmographic (PPG) signals recorded from the faces of study participants were converted to the YCgCr color space. Substituting the peaks and valleys extracted from the converted Cg and Cr PPG signals into the Beer–Lambert law yields the SPO_2_ via a linear equation. When the non-contact SPO_2_ measurement value was evaluated based on the reference SPO_2_ measured with a pulse oximeter, the mean absolute error was 0.537, the root mean square error was 0.692, the Pearson correlation coefficient was 0.86, the cosine similarity was 0.99, and the intraclass correlation coefficient was 0.922. These results confirm the feasibility of non-contact SPO_2_ measurements.

## 1. Introduction

Blood oxygen saturation (SPO_2_) refers to the concentration of oxygenated hemoglobin relative to the total amount of hemoglobin in the blood. A normal blood oxygen saturation level is 95–100% at sea level and falls below 90% due to hypoxemia or other reasons. Severe hypoxemia (SPO_2_ < 80%) can have serious implications for the brain, heart, and lungs, and requires immediate attention [1]. In addition, COVID-19 patients who often show low SPO_2_ (SPO_2_ < 90%) are hospitalized, and SPO_2_ has been used as an indicator in the diagnosis of COVID-19 [2]. SPO_2_ is an important indicator of health and can be used in the early-stage detection of respiratory diseases. The current standard for SPO_2_ measurement is pulse oximetry using the photoplethysmographic (PPG) method, which measures changes in blood volume through the amount of light transmitted or reflected after irradiating the skin with light. Using this measurement method and the fact that oxygenated hemoglobin and deoxygenated hemoglobin absorb red and infrared light differently, the sensor is contacted with the patient’s body, typically the finger or ear lobe [3]. However, this method requires infrared and red-light illumination and contact with the sensor, with the latter potentially causing discomfort and skin irritation. Moreover, when sensors are attached to body parts, participants can consciously or unconsciously influence oxygen saturation values because they are aware that they are being monitored. Therefore, in this study, we propose a non-contact method for measuring SPO_2_.

Previous studies involving photoplethysmographic (PPG)-based SPO_2_ measurements have proposed using light sources with specific wavelengths and multiple cameras. For example, Kong et al. proposed an SPO_2_ measurement method that uses light with wavelengths of 520 nm and 660 nm. To use two wavelengths of light, the signals were extracted by photographing faces through two cameras with narrow-pass filters. After determining the alternating current (AC)/direct current (DC) signal ratio at both wavelengths, the SPO_2_ was measured through calibration [4]. Tamura also used the ratio method, but with 660 nm (red) and 940 nm (infrared) light sources [5]. However, in this paper, we propose a non-contact SPO_2_ measurement technique that uses ambient light through a single RGB camera without requiring additional illumination.

In order to measure SPO_2_ in a non-contact manner, it is necessary to measure a heart signal, that is, a PPG signal, in a non-contact manner. In general, the PPG signal is obtained by attaching a sensor to the skin and measuring the amount of transmitted light by emitting light. However, this method has the inconvenience of having to attach a sensor to the body. Therefore, studies on remote PPG (rPPG), a technology for measuring PPG signals in a non-contact manner using a camera, are being conducted. Verkruysse et al. proposed a method for measuring heart rate using ambient light and a digital camera and showed that the green channel contains the strongest plethysmography signal because hemoglobin absorbs green light the most, and green light can penetrate deep into the skin. In addition, it was shown that the red and blue channels also contain plethysmography information [6]. In the paper of Poh et al., it was explained that the RGB color sensor is mixed with the original signal with different weights for each color because the absorption rate of hemoglobin is different at each wavelength. Therefore, using Independent Component Analysis (ICA), the color channels were separated into independent components in the RGB image of the face, and the heart rate was extracted through this method [7]. Song et al. proposed an rPPG method showing excellent performance against noise by mapping between spatiotemporal heart rate (HR) feature images and corresponding HR values using CNN [8]. These existing rPPG technologies are focused on calculating heart rate rather than extracting rPPG raw signals, as it is difficult to measure SPO_2_ using rPPG raw signals. Recently, Allado et al. proposed a method to obtain physiological variables such as heart rate, respiratory, and oxygen saturation by obtaining raw data through red, green, and blue color spectra measurement from face images [9]. Like this study, research on raw data analysis has been conducted recently, but detailed algorithms for extracting vital signs such as oxygen saturation using raw data and quality of raw signals are unknown.

Unlike previous studies, lab-made rPPG technology can extract rPPG raw signals in real time, so bio-markers such as HR and SPO_2_ can be extracted from this signal. Since this technology extracts the rPPG signal from the color image, SPO_2_ can be obtained using this RGB raw signal. Therefore, it is possible to implement a non-contact system capable of SPO_2_ analysis in real time beyond the level of rPPG technology that simply extracts heart rate.

We can extract the raw PPG signal in a non-contact manner, which is essential for measuring oxygen saturation in a non-contact manner. In addition, this method does not cause inconvenience when measuring oxygen saturation in a contact manner. Therefore, this paper proposes a method for measuring oxygen saturation in a non-contact manner. The remainder of this paper proceeds as follows. Section 2 explains the SPO_2_ measurement principle, PPG signal extraction, and the SPO_2_ measurement method. In Section 3, the results are analyzed by comparing SPO_2_ measurements acquired using the conventional sensor-based method with those acquired using our proposed method. Section 4 analyzes the results and describes the need for further research. Finally, Section 5 presents the research conclusion and future research.

## 2. Methods

### 2.1. Oxygen Saturation Measurement Principle

**Figure 1:**
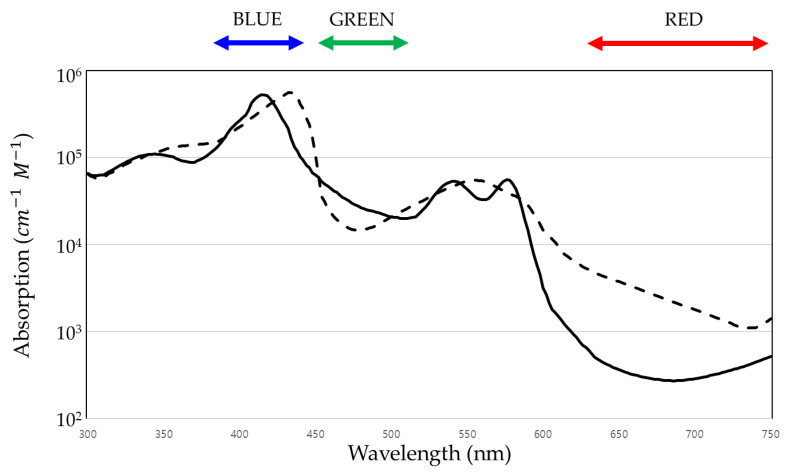
Wavelength-dependent absorption coefficients of oxygenated (solid line) and deoxygenated hemoglobin (dashed line).

Camera-based SPO_2_ measurements use PPG and the Beer–Lambert law. In PPG, blood flow is determined according to the amount of incident light that is reflected or transmitted [10]. Blood flow affects not only the reflectance but also the type and concentration of substances in the blood. As shown in Figure 1, in the case of green light, oxygenated hemoglobin absorbs green light more strongly than deoxygenated hemoglobin. On the other hand, in the case of red light, deoxygenated hemoglobin absorbs red light more strongly than oxygenated hemoglobin [11]. This wavelength-dependent behavior of hemoglobin absorption can be used to measure SPO_2_. In addition, PPG signals contain both an alternating current (AC), a pulsatile waveform caused by heartbeats, and a direct current (DC), a non-pulsatile waveform caused by veins, other tissues, artifacts, and the respiratory modulation [12]. Since SPO_2_ is measured using pulse changes in the arteries, we use an AC component.

The Beer–Lambert law states that the absorption of light is proportional to the concentration of the substance and the optical penetration depth [13]. It is expressed as:

(1) $${I=I}_{0}e^{-\varepsilon (\lambda )\mathrm{Cl}},$$

where I is the reflected light intensity, $I_{0}$ is the incident light intensity, ε(λ) is the absorption coefficient at wavelength λ, C is the concentration, and l is the optical path length. Using Equation (1), the concentration of oxygenated hemoglobin and deoxygenated hemoglobin in the blood can be obtained through the intensity of reflected light. The SPO_2_ is calculated by using this law and the AC component of the PPG.

### 2.2. Non-Contact SPO_2_ Measurement Method

**Figure 2:**
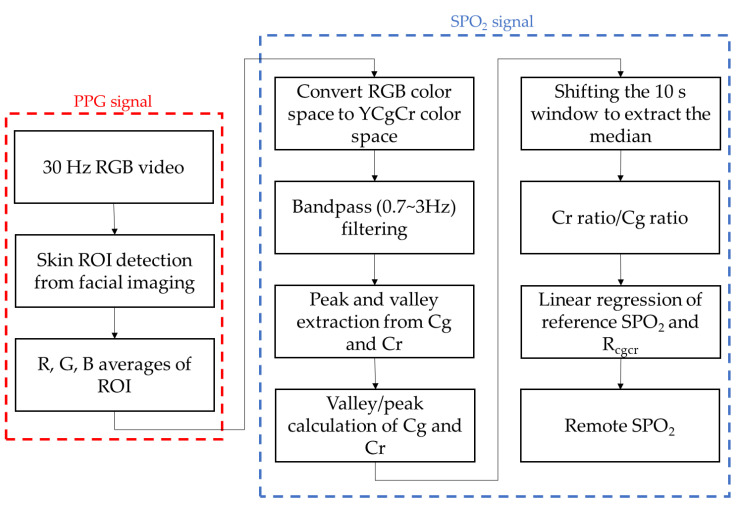
Non-contact oxygen saturation measurement process. R = red; B = blue; G = green; ROI = region of interest; R_cgcr_ = Cr ratio/Cg ratio.

The proposed non-contact SPO_2_ measurement process is shown in Figure 2. The PPG signal is extracted from the camera and the SPO_2_ is calculated based on the extracted signal. To extract the PPG signal from the camera, face image data were captured for 5 min using an RGB camera [14]. At the same time, reference SPO_2_ data were obtained by attaching a CMS-50E [15] pulse oximeter to the participant’s finger. During the experiment, hypoxia was induced by instructing participants to hold their breath for 1 min, reducing SPO_2_ by an average of 10%. A total of 10 healthy Koreans (5 males and 5 females) aged 23 to 31 years took part in the study. Before the experiment, subjects were instructed to minimize finger and facial movements and not to put on makeup to prevent noise. The experiment was conducted in an indoor environment with an average temperature of 20 to 24 °C, average humidity of 50% to 60%, and an average brightness of 500 lux. The research followed the principle of the Declaration of Helsinki, and informed consent was obtained from the subjects after an explanation of nature.

The process of extracting the PPG signal is explained in Section 2.2.1, while the process of determining the SPO_2_ from the extracted signal is described in Section 2.2.2.

#### 2.2.1. Remote PPG Signal

**Figure 3:**
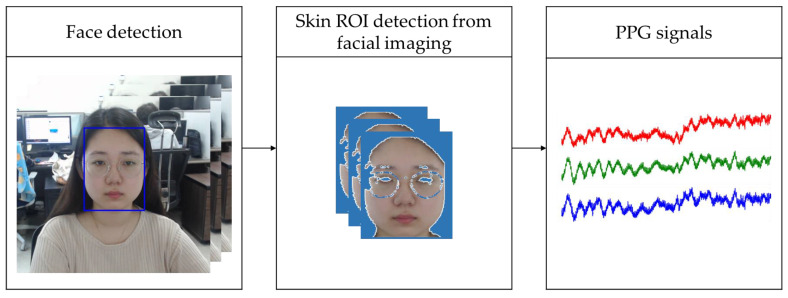
PPG signal acquisition process. The red line is the R raw signal, the green line is the G raw signal, and the blue line is B raw signal.

Non-contact SPO_2_ measurements necessitate non-contact PPG signal measurements. To obtain a non-contact PPG signal, we used lab-made remote-PPG (rPPG) technology developed in-house that can measure PPG signals in real time using a camera [16]. Specifically, our lab-made system measures the PPG signal according to blood flow-induced changes in skin color. This technique obtains PPG signals as follows. First, a face is detected in an RGB image frame at 30 frames per second and then tracked in a continuous image frame using a kernelized correlation filter (KCF) tracker [17]. To observe changes in skin color, a specific region of skin on the detected face is selected as the region of interest (ROI). In this case, if a face region excluding the background is simply selected, the intensity of the blood flow signal is minute, and the signal may be distorted due to noise caused by lighting changes and artifacts. Therefore, after converting the image frame from the RGB color space to the YCbCr color space, skin pixel clustering is performed in the Cb-Cr plane to utilize the skin pixel characteristics. The ROI of the selected face region means the entire skin area of the face except for eyebrows, eyes, and accessories such as glasses. This is because if the ROI is small, it will be greatly affected by noise, such as the natural tremor of a person, and if the ROI is the forehead, there is a risk that it will be covered with hair. Next, the PPG signal is extracted by calculating the average of each of R, G, and B in the selected ROI of each frame. The rPPG signal extraction process is summarized in Figure 3, and the real-time rPPG signal extraction can be viewed in the Supporting Information of the video by [18].

#### 2.2.2. SPO_2_ Signal

##### SPO_2_ Measurement Using YCgCr Signal

The RGB color space is converted to the YCgCr color space by applying Equation (2) [19] to the PPG signal extracted in Section 2.2.1. Y is the luminance component, and Cg and Cr are the green-difference and red-difference chroma components, respectively. In Equation (2), R′, G′, and B′ represent the values of R, G, and B, respectively, normalized to a value between 0 and 1 by dividing by the maximum pixel value of 255:

(2) $$\begin{array}{c} Y=16\;+\;\left(65{.481\;\times \;R}^{\prime }\right)+\left(128{.533\;\times \;G}^{\prime }\right)+\left(24{.966\;\times \;B}^{\prime }\right)\\ \mathrm{Cg}=128\;+\;\left(-81{.085\;\times \;R}^{\prime }\right)+\left({112\;\times G}^{\prime }\right)+\left(-30{.915\;\times B}^{\prime }\right)\\ \mathrm{Cr}=128\;+\;\left({112\;\times \;R}^{\prime }\right)+\left(-93{.786\;\times \;G}^{\prime }\right)+\left(-18{.214\;\times \;B}^{\prime }\right) \end{array}$$

SPO_2_ is measured using two or more wavelengths of light and utilizing the different absorption characteristics of different types of hemoglobin. At green and red wavelengths, the absorption of oxygenated and deoxygenated hemoglobin exhibit opposite responses. Therefore, we chose to use these wavelengths for our study. The RGB color space was converted to the YCgCr color space to eliminate the effect of light brightness and use these green and red wavelengths. To extract the heartbeat-related signals covering the entire span of the expected physiological heart rate range from the converted Cg and Cr signals, bandpass filtering was applied to pass only frequencies ranging 0.7–3 Hz (corresponding to 42 to 180 beats/min). In this case, bandpass filtering was performed using a zero-phase five-order Butterworth filter [20]. Based on our use of brightness-independent Cg and Cr signals, the Beer–Lambert law in Equation (1) was modified to Equation (3) to calculate the SPO_2_:

(3) $${I=e}^{-\varepsilon (\lambda )\mathrm{Cl}}.$$

In the filtered AC signals of Cg and Cr, the maximum (minimum) amplitudes are denoted as $I_{\mathrm{cg},p}$ and $I_{\mathrm{cr},p}$ ($I_{\mathrm{cg},v}$ and $I_{\mathrm{cr},v}$), respectively. In accordance with Equation (3), this can be expressed as:

(4) $$I_{\mathrm{cg},p}{=e}^{-\left[\varepsilon_{\mathrm{Hb}}\left(\lambda_{\mathrm{cg}}\right)C_{\mathrm{Hb}}{+\varepsilon }_{{\mathrm{HbO}}_{2}}\left(\lambda_{\mathrm{cg}}\right)C_{{\mathrm{HbO}}_{2}}\right]l},$$

(5) $$I_{\mathrm{cg},v}{=e}^{-\left[\varepsilon_{\mathrm{Hb}}\left(\lambda_{\mathrm{cg}}\right)C_{\mathrm{Hb}}{+\varepsilon }_{{\mathrm{HbO}}_{2}}\left(\lambda_{\mathrm{cg}}\right)C_{{\mathrm{HbO}}_{2}}\right]\left(l+∆l\right)},$$

(6) $$I_{\mathrm{cr},p}{=e}^{-\left[\varepsilon_{\mathrm{Hb}}\left(\lambda_{\mathrm{cr}}\right)C_{\mathrm{Hb}\;}{+\varepsilon }_{{\mathrm{HbO}}_{2}}\left(\lambda_{\mathrm{cr}}\right)C_{{\mathrm{HbO}}_{2}}\right]l},$$

(7) $$I_{\mathrm{cr},v}{=e}^{-\left[\varepsilon_{\mathrm{Hb}}\left(\lambda_{\mathrm{cr}}\right)C_{\mathrm{Hb}}{+\varepsilon }_{{\mathrm{HbO}}_{2}}\left(\lambda_{\mathrm{cr}}\right)C_{{\mathrm{HbO}}_{2}}\right]\left(l+∆l\right)},$$

where $\varepsilon_{\mathrm{Hb}}\left(\lambda_{\mathrm{cg}}\right)C_{\mathrm{Hb}}$ and $\varepsilon_{\mathrm{Hb}}\left(\lambda_{\mathrm{cr}}\right)C_{\mathrm{Hb}}$ are the products of the absorption coefficient and concentration of deoxygenated hemoglobin at the Cg and Cr wavelengths. $\varepsilon_{{\mathrm{HbO}}_{2}}\left(\lambda_{\mathrm{cg}}\right)C_{{\mathrm{HbO}}_{2}}$ and $\varepsilon_{{\mathrm{HbO}}_{2}}\left(\lambda_{\mathrm{cr}}\right)C_{{\mathrm{HbO}}_{2}}$ are the products of the absorption coefficient and concentration of oxygenated hemoglobin at the Cg and Cr wavelengths. l and ∆l are the light penetration depth in the arteries, which indicates the volume of blood. l means the thickness of the thinnest arterial blood vessel, that is, when the blood flow is the minimum. ∆l is the change in blood flow due to heartbeat. Because the volume of blood and the reflected light intensity are inversely proportional, the reflected light intensity corresponding to the smallest blood volume is used to calculate the peak point in Equations (4) and (6). In addition, when calculating the valley point in Equations (5) and (7), l+∆l, which represents the case of increased blood volume, is used. The SPO_2_ can be measured in the part that fluctuates with the heartbeat. Therefore, as in Equations (8) and (9), only the effect of hemoglobin is left in the part that is changed by the heartbeat through the logarithm of the ratio of peak to valley.

(8) $$\ln\left(\frac{I_{\mathrm{cg},v}}{I_{\mathrm{cg},p}}\right)=-\left[\varepsilon_{\mathrm{Hb}}\left(\lambda_{\mathrm{cg}}\right)C_{\mathrm{Hb}}{+\varepsilon }_{{\mathrm{HbO}}_{2}}\left(\lambda_{\mathrm{cg}}\right)C_{{\mathrm{HbO}}_{2}}\right]∆l,$$

(9) $$\ln\left(\frac{I_{\mathrm{cr},v}}{I_{\mathrm{cr},p}}\right)=-\left[\varepsilon_{\mathrm{Hb}}\left(\lambda_{\mathrm{cr}}\right)C_{\mathrm{Hb}}{+\varepsilon }_{{\mathrm{HbO}}_{2}}\left(\lambda_{\mathrm{cr}}\right)C_{{\mathrm{HbO}}_{2}}\right]∆l.$$

**Figure 4:**
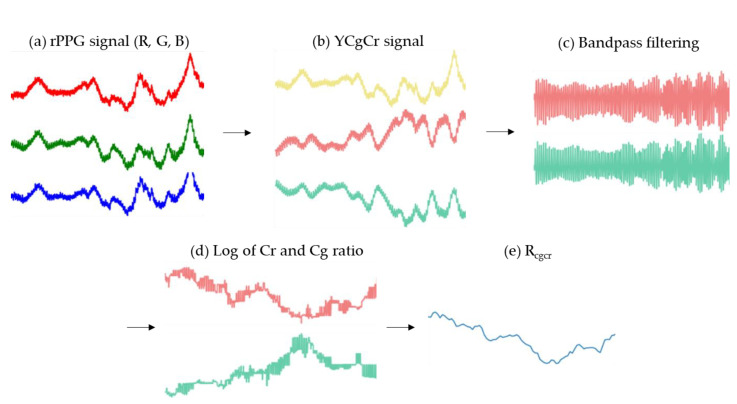
Process of extracting *R_cgcr_* value from rPPG signal (**a**) R, G, B signals extracted from face ROI. The red line is the R raw signal, the green line is the G raw signal, and the blue line is B raw signal. (**b**) R, G, B signals are converted into YCgCr color space. The yellow line is the Y signal, the pink line is the Cr signal, and the light blue line is the Cg signal. (**c**) Result of bandpass filtering on Cg and Cr signals. The pink line is the filtered Cr signal, the light blue line is the filtered Cg signal. (**d**) The result of Equations (8) and (9). That is, the log value of the peak/valley extracted from the Cg and Cr signals to which bandpass filtering is applied, respectively. The pink line is the calculated Cr signal, the light blue line is the calculated Cg signal. (**e**) The result of Equation (10), that is, Cr ratio/Cg ratio.

Then, to reduce noise caused by other factors excepting for oxygen saturation, the logarithmic function was applied in such a way that the median value was extracted when the 10-s window was moved at one sample interval. However, the effect of fluctuating blood volume remains. As shown in Figure 4d, it can be confirmed that the Cg and Cr ratios reflect changes in SPO_2_ but contain a large number of high-frequency noise components due to heartbeat. To eliminate this effect, the ratio of two different wavelengths is obtained:

(10) $$R_{\mathrm{cgcr}}=\frac{\ln\left(\frac{I_{\mathrm{cr},v}}{I_{\mathrm{cr},p}}\right)}{\ln\left(\frac{I_{\mathrm{cg},v}}{I_{\mathrm{cg},p}}\right)}=\frac{\varepsilon_{\mathrm{Hb}}\left(\lambda_{\mathrm{cr}}\right)C_{\mathrm{Hb}}{+\varepsilon }_{{\mathrm{HbO}}_{2}}\left(\lambda_{\mathrm{cr}}\right)C_{{\mathrm{HbO}}_{2}}}{\varepsilon_{\mathrm{Hb}}\left(\lambda_{\mathrm{cg}}\right)C_{\mathrm{Hb}}{+\varepsilon }_{{\mathrm{HbO}}_{2}}\left(\lambda_{\mathrm{cg}}\right)C_{{\mathrm{HbO}}_{2}}}.$$

As a result, only the effect of hemoglobin according to the wavelength remains, and SPO_2_ can be obtained. Note that R_cgcr_ was calibrated using the linear regression between the obtained R_cgcr_ value and the reference SPO_2_ level.

##### SPO_2_ Measurement Using YCbCr Signal

As shown in Figure 1, there is a part where the absorption coefficients of non-oxygenated hemoglobin and oxygenated hemoglobin are opposite to each other at blue and red wavelengths. Therefore, oxygen saturation can be measured using blue and red wavelengths. To apply this fact, after converting the RGB color space of the rPPG signal to YCbCr, the blue-difference chroma component Cb and the red-difference chroma component Cr signal are extracted. After calculating the extracted Cb and Cr signals as described above to obtain the R_cbcr_ value and performing linear regression with the reference SPO_2_, SPO_2_ can be obtained by calibrating these values.

## 3. Results

### 3.1. Correlation between R_cgcr_ Value Obtained Using YCgCr Signal and Reference SPO_2_

**Figure 5:**
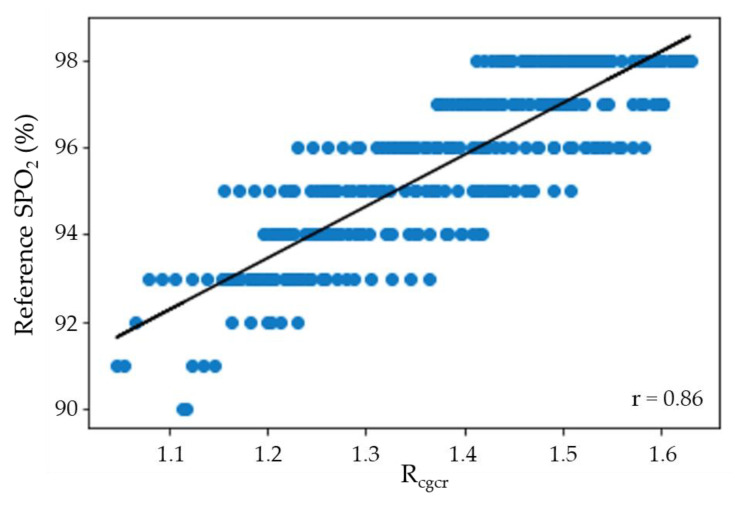
Scatter plot showing the relationship between R_cgcr_ and the reference SPO_2_.

The R_cgcr_ values were obtained from the face image recorded using the method described in Section 2.2. The R_cgcr_ values were calibrated to obtain the remote SPO_2_ (rSPO_2_). However, prior to this calibration, the Pearson correlation coefficient was calculated to ascertain whether a linear relationship existed between the R_cgcr_ value and the reference SPO_2_. As shown in Figure 5, a Pearson’s correlation coefficient of 0.86 was determined, confirming the linear correlation between the R_cgcr_ value and the reference SPO_2_. The reason blue dots appear as horizontal lines in Figure 5 is that the reference SPO_2_ value is measured as an integer, but the R_cgcr_ value is measured as a continuous real number.

After this linear relationship was confirmed, the R_cgcr_ values were derived from Equation (11) through linear regression with the reference SPO_2_ and corrected for ${\mathrm{rSPO}}_{2}^{\mathrm{cgcr}}$.

(11) $${\mathrm{rSPO}}_{2}^{\mathrm{cgcr}}=11.8805\;\times \;\mathrm{Rcgcr}+79.1914.$$

### 3.2. Correlation between Rcbcr Value Obtained Using YCbCr Signal and Reference SPO_2_

The R_cbcr_ value was obtained by converting the RGB color space of the rPPG signal to YCbCr. Now, by calibrating this R_cbcr_ value, ${\mathrm{rSPO}}_{2}^{\mathrm{cbcr}}$ can be obtained. Before calibration, Pearson’s correlation coefficient was obtained to check whether there is any relationship between the R_cbcr_ value and the reference SPO_2_. As a result, 0.27 was obtained, and the following Equation (12) was derived through linear regression with reference SPO_2_.

(12) $${\mathrm{rSPO}}_{2}^{\mathrm{cbcr}}=7.40325\;\times \;\mathrm{Rcgcr}+87.9765.$$

### 3.3. Comparison of rSPO_2_ and Reference SPO_2_

**Figure 6:**
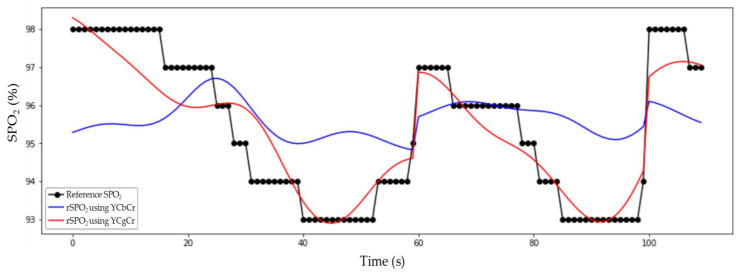
Comparison of rSPO_2_ obtained by non-contact and reference SPO_2_ obtained by contact sensor. The black line is the reference SPO_2_, the red line is the ${\mathrm{rSPO}}_{2}^{\mathrm{cgcr}}$, and the blue line is the ${\mathrm{rSPO}}_{2}^{\mathrm{cbcr}}$.

Figure 6 compares rSPO_2_ measured using CgCr and rSPO_2_ measured using CbCr with reference SPO_2_. It can be confirmed that rSPO_2_ measured using Cg and Cr was better estimated when compared with reference SPO_2_ than rSPO_2_ measured using Cb and Cr. However, to confirm this quantitatively, mean absolute error (MAE), root mean squared error (RMSE), cosine similarity, and intraclass correlation coefficient (ICC) [21] were used. These results can be seen in Table 1. MAE and RMSE for calculating the distance between reference SPO_2_ and rSPO_2_ are 0.537 and 0.692 for ${\mathrm{rSPO}}_{2}^{\mathrm{cgcr}}$, respectively, which are smaller than 1.547 and 1.754 for ${\mathrm{rSPO}}_{2}^{\mathrm{cbcr}}$. This indicates that the absolute difference between ${\mathrm{rSPO}}_{2}^{\mathrm{cgcr}}$ and reference SPO_2_ is small, indicating that ${\mathrm{rSPO}}_{2}^{\mathrm{cgcr}}$ was predicted better than ${\mathrm{rSPO}}_{2}^{\mathrm{cbcr}}$. In the ICC 95% confidence interval, ${\mathrm{rSPO}}_{2}^{\mathrm{cgcr}}$ was 0.922 and ${\mathrm{rSPO}}_{2}^{\mathrm{cbcr}}$ was 0.243. This means that in the case of ${\mathrm{rSPO}}_{2}^{\mathrm{cgcr}}$, it is 92.2% identical to the reference SPO_2_, and in the case of ${\mathrm{rSPO}}_{2}^{\mathrm{cbcr}}$, it is 24.3% identical. At this time, in the case of ${\mathrm{rSPO}}_{2}^{\mathrm{cgcr}}$, it is statistically significant because the significance probability is less than 0.001. The cosine similarity shows how similar the directions of the two vectors are, and 0.999 was obtained for both ${\mathrm{rSPO}}_{2}^{\mathrm{cgcr}}\;$ and ${\mathrm{rSPO}}_{2}^{\mathrm{cbcr}}$. It can be seen that when the reference SPO_2_ decreases, rSPO_2_ also decreases, and vice versa. However, as shown in Figure 6, in the case of ${\mathrm{rSPO}}_{2}^{\mathrm{cbcr}}$, the trend may be similar, but the error is large, so MAE and RMSE should be considered together to determine whether rSPO_2_ is well predicted. Therefore, as shown in the statistical results, it can be seen that ${\mathrm{rSPO}}_{2}^{\mathrm{cgcr}}$ predicted SPO_2_ well.

## 4. Discussion

In this paper, we proposed a method to measure oxygen saturation in a non-contact manner by extracting a remote PPG signal. There are two methods for measuring SPO_2_ in a non-contact manner, one using the Cg and Cr signal and the other using the Cb and Cr signal. Consequently, the use of Cg and Cr signals is recommended. This study measures SPO_2_ using an RGB camera in ambient light, which has a broadband spectral response rather than a narrowband spectral response. This is because the absorption coefficients of the two hemoglobin are opposite at the blue and red wavelengths only in the narrowband of blue. Therefore, the method using the Cb and Cr signal does not utilize the characteristic that the absorption coefficients of non-oxygenated hemoglobin and oxygenated hemoglobin are opposite at different wavelengths, so the results are not good.

There are several factors that can be improved in the future. We calculated the oxygen saturation from the raw R, G, and B signals in the rPPG signal. This raw signal contains motion artifacts and noise, but no other processing than frequency filtering is performed, so noise such as motion may affect the quality of the signal. Therefore, if motion information is obtained through the camera and additional pre-processing is performed, a motion robust signal can be obtained. Furthermore, this study involved SPO_2_ levels of 85–100% because the SPO_2_ value was lowered deliberately by instructing participants to hold their breath. In the future, it is necessary to further lower the SPO_2_ level and develop a proposed method to broaden the measurement range of the non-contact method.

## 5. Conclusions

This paper describes a technique for non-contact SPO_2_ measurements using a single RGB camera under ambient light conditions. In this study, we utilized the opposite absorption characteristics of hemoglobin at the green and red wavelengths. Consequently, the Cg and Cr signals were extracted by converting the RGB color space of the PPG signal extracted from facial images to the YCgCr color space. After obtaining AC signals from each of the extracted Cg and Cr signals, find the peak and valley points and take the logarithm of the peak/valley ratio. These values move the 10-s window at 1-sample intervals and extract the median value, and calculate the R_cgcr_ value through the Cr/Cg ratio. The rSPO_2_ was obtained by applying the linear regression-extracted equation to the resultant R_cgcr_ value. Comparing the measured ${\mathrm{rSPO}}_{2}^{\mathrm{cgcr}}$ with the reference SPO_2_ data, the MAE was 0.537, the RMSE was 0.692, the Pearson’s correlation coefficient was 0.86, the cosine similarity was 0.99, and the ICC was 0.922. These results indicate that the SPO_2_ measured by the proposed non-contact method is consistent with SPO_2_ measurements acquired via conventional methods involving the attachment of a sensor, thus confirming the feasibility of accurate non-contact SPO_2_ measurements. In future works, we plan to conduct a wide range of oxygen saturation measurement studies using critically ill patient data for clinical research. In other words, by extending the value range of SPO_2_ ground-truth, the correlation with rPPG will be more clearly identified. In addition, further studies will be conducted to improve the speed of extraction of rPPG signals to obtain oxygen saturation.

## Figures and Tables

**Table 1:** Statistical results of ${\mathrm{rSPO}}_{2}^{\mathrm{cgcr}}$ and ${\mathrm{rSPO}}_{2}^{\mathrm{cbcr}}$. MAE = mean absolute error; RMSE = root mean squared error.

	**MAE**	**RMSE**	**Cosine Similarity**	**Intraclass Correlation Coefficient**		
				**Intraclass Correlation**	**95% Confidence Interval**	**Sig**
${\mathrm{rSPO}}_{2}^{\mathrm{cgcr}}$	0.537 (±0.436)	0.692	0.999	0.922	0.905–0.936	0.000
${\mathrm{rSPO}}_{2}^{\mathrm{cbcr}}$	1.547 (±0.827)	1.754	0.999	0.243	0.075–0.381	0.003

## Data Availability

The obtained data cannot be shared because it was agreed that they could be used only for this study.
